# PKM2 is involved in neuropathic pain by regulating ERK and STAT3 activation in rat spinal cord

**DOI:** 10.1186/s10194-018-0836-4

**Published:** 2018-01-18

**Authors:** Binbin Wang, Siyuan Liu, Bingbing Fan, Xingguo Xu, Yonglin Chen, Rongxiang Lu, Zhongling Xu, Xiaojuan Liu

**Affiliations:** 1 Department of Anesthesiology, Affiliated Hospital of Nantong University, Nantong, Jiangsu 226001 China; 2 Department of Anesthesiology, Nantong Maternity and Child Health Hospital, Nantong, Jiangsu 226018 China; 3 Department of Radiology, Zhongshan Hospital, Fudan University, Shanghai Institute of Medical Imaging, Department of Medical Imaging, Shanghai Medical College, Fudan University, Shanghai, 200032 China; 4 Department of Pathogen Biology, Medical College, Nantong University, Nantong, Jiangsu 2266001 China

**Keywords:** PKM2, Neuropathic pain, Chronic constriction injury, Lactate, p-ERK, p-STAT

## Abstract

**Background:**

Pyruvate kinase isozymes M2 (PKM2), as a member of pyruvate kinase family, plays a role of glycolytic enzyme in glucose metabolism. It also functions as protein kinase in cell proliferation, signaling, immunity, and gene transcription. In this study, the role of PKM2 in neuropathic pain induced by chronic constriction injury (CCI) was investigated.

**Methods:**

Rats were randomly grouped to establish CCI models. PKM2, extracellular regulated protein kinases (EKR), p-ERK, signal transducers and activators of transcription (STAT3), p-STAT3, phosphoinositide 3-kinase/protein kinase B (PI3K/AKT) and p-PI3K/AKT proteins expression in spinal cord was examined by Western blot analysis. Cellular location of PKM2 was examined by immunofluorescence. Knockdown of PKM2 was achieved by intrathecal injection of specific small interfering RNA (siRNA). Von Frey filaments and radiant heat tests were performed to determine mechanical allodynia and thermal hyperalgesia respectively. Lactate and adenosine triphosphate (ATP) contents were measured by specific kits. Tumor necrosis factor alpha (TNF-α) and interleukin-1 beta (IL-1β) levels were detected by ELISA kits.

**Results:**

CCI markedly increased PKM2 level in rat spinal cord. Double immunofluorescent staining showed that PKM2 co-localized with neuron, astrocyte, and microglia. Intrathecal injection of PKM2 siRNA not only attenuated CCI-induced ERK and STAT3 activation, but also attenuated mechanical allodynia and thermal hyperalgesia induced by CCI. However, PKM2 siRNA failed to inhibit the activation of AKT. In addition, PKM2 siRNA significantly suppressed the production of lactate and pro-inflammatory mediators.

**Conclusion:**

Our findings demonstrate that inhibiting PKM2 expression effectively attenuates CCI-induced neuropathic pain and inflammatory responses in rats, possibly through regulating ERK and STAT3 signaling pathway.

## Background

Neuropathic pain, which is caused by heterogeneous etiology, remains a Gordian knot for pain management practitioners [1]. Despite numerous investigations focused on nociceptors, modulators and downstream signaling pathways in the past few decades [2, 3], we still can’t elucidate the underlying mechanisms of neuropathic pain very well.

According to the agreed definition, issued by the International Association for the Study of Pain, neuropathic pain is caused by a lesion or disease of the somatosensory nervous system. The lesion or dysfunction results in the localized release of neurotransmitters, neurotrophic factors, cytokines and chemokines. These substances increase the sensitivity and excitability of primary sensory neurons by lowering the activation threshold of peripheral nociceptors, which results in peripheral sensitization [4, 5]. Increased outputs from primary afferent terminals triggers synaptic plasticity and long lasting transcriptional and post-translational changes in central nervous system, which is defined as central sensitization [6, 7]. Peripheral and central sensitization are considered important mechanism in neuropathic pain and contribute to hypersensitive pain behaviors [7]. Inflammatory process and metabolic dysregulation are two sides of the same coin in central sensitization [8, 9]. After peripheral nerve injury, glial cells are initially activated and subsequently generate numerous pro-inflammatory mediators, contributing to the development of neuropathic pain [10, 11]. The pro-inflammatory phenotype of glial cells request enhanced energy supply [12, 13], displaying a glycolytic metabolic shift from oxidative phosphorylation to aerobic glycolysis [14]. The enhanced glycolysis in cells provides biosynthetic precursors for pro-inflammatory proteins [15].

Glucose metabolism is precisely regulated by a number of glycolytic enzymes, including hexokinases, pyruvate kinase, and pyruvate dehydrogenase. Pyruvate kinase is a rate limiting enzyme catalyzing the final step of glycolysis, converting phosphoenolpyruvic acid and ADP to pyruvate and ATP [16]. PKM2 is one of the four pyruvate kinase isoforms, and mainly expresses in normal proliferating cells and cancer cells [16]. Under catalysis of PKM2, glucose entering glycolytic pathway is metabolized to lactate and ATP rather than oxidized in mitochondria [17]. ATP, ligand of P2X family receptor, is an important pain mediator [18]. Lactate is an energy substrate for neurons [19] and has been identified as an important signaling molecule in neuro-immune, neuronal plasticity, neuron-glia interactions, as well as nociception [9, 20–22]. Lactate can also furtherly promote glial cells to release the pro-inflammatory cytokines under pathological conditions [23]. With the deepening of research, PKM2 is found to be a generalist, which can also function as a protein kinase by nuclear translocation under pathological stimulation [24]. PKM2 can phosphorylates ERK1/2, STAT-3 and PI3K/AKT, enhancing cell proliferation and subsequent related gene transcription [25–27]. These signaling pathways are activated in spinal cord (SC) after nerve injury and contribute to the development of neuropathic pain [28–30]. PKM2 also interacts with hypoxia-inducible factor 1-alpha(HIF-1α) and upregulates the expression of IL-1β [31] in LPS-activated macrophages. Although PKM2 plays an important role in metabolism, gene transcription and inflammation, whether or not it participates in the process of neuropathic pain remains unknown.

In this study, we developed a clinically relevant model of neuropathic pain induced by CCI and investigated the potential role of PKM2 in neuropathic pain. We were confirmed that CCI induced significant upregulation of PKM2 in SC. We also demonstrated that inhibiting PKM2 by intrathecal injection of specific siRNA effectively attenuated CCI-induced rat neuropathic pain and inflammatory responses, possibly through regulating ERK and STAT3 signaling pathway. This research may represent a novel strategy for treating neuropathic pain.

## Methods

### Animals

Male Sprague-Dawley rats weighed 200–240 g were obtained from Experimental Animal Center, Nantong University. Rats were housed on a 12:12 light-dark cycle at 22 ± 1 °C with free access to food and water. The experimental procedures were approved by the Animal Care and Use Committee of Nantong University and were conducted in accordance with guidelines of the International Association for the Study of Pain.

### Establishment of the neuropathic pain model

CCI model of sciatic nerve injury was established according to procedures described by Ding Y et al. [32]. In brief, rats were anesthetized with isoflurane, and the left sciatic nerve was loosely ligated by 4-0 chromic gut sutures at four segments with 1 mm apart. The sutures were gently tightened until a brisk twitch of the left hind limb was observed. The same surgical procedure was performed in the sham groups except ligating the sciatic nerve.

### Behavioral testing

Mechanical allodynia was measured by von Frey filaments. Rats were placed into a transparent plexiglas compartments upon a metal mesh. The plantar surface of left paw was perpendicularly subjected to a series of von Frey hairs with logarithmically incrementing stiffness (0.4-26 g) until the filaments bowed slightly. Rapid paw withdrawal or flinching were considered positive responses. The 50% paw withdrawal threshold (PWT) was determined using Dixon’s up-down method [33]. Thermal hyperalgesia was tested by radiant heat using Hargreaves apparatus (IITC Life Science Inc., Woodland Hills, CA) and represented as paw-withdrawal latency (PWL). The rats were placed in hyaline plastic compartments and the plantar surface of the left hind paw was exposed to a radiant heat source through the glass plate. Mean PWL was averaged from latency of three successive tests. A cut-off time of 20s was set to prevent tissue damage.

### PKM-2 siRNA and lumbar intrathecal injection

The siRNA was commercially synthesized (Genepharma, Shanghai, China). siRNA duplexes that specifically targeted PKM2 were: sense 5’-CAUCUACCACUUGCAAUUATT-3′ and anti-sense 5′- UAAUUGCAAGUGGUAGAUGTT-3′. Non-targeting siRNA (NT-siRNA) was synthesized by a scrambled sequence of nucleotides as a control siRNA. Before intrathecal injection, siRNA was dissolved by RNase-free water to a concentration of 0.75μg/μl and then mixed with polyethyleneimine (PEI, dissolved in 5% glucose, 1 μg of siRNA was mixed with 0.18 μl of PEI) for 10 min. Intrathecal injection was performed with a micro syringe between L5 and L6 intervertebral spaces to deliver the reagents (20 μl) into the cerebral spinal fluid [34]. Once the needle inserted subarachnoid space successfully, a brisk tail flick could be observed.

### Western blot analysis

Lumbar SC (L4–L5) were excised rapidly from deeply anesthetized rats. Total proteins were extracted by protein extraction kits, separated by 8% SDS–PAGE and then transferred onto a PVDF membrane. The membranes were blocked with 5% milk and incubated overnight at 4 °C with primary antibody against PKM-2 (anti-mouse, 1:500, Santa Cruz, USA), Stat3 (anti-mouse, 1:500, Santa Cruz, USA), p-Stat3 (anti-mouse, 1:500, Santa Cruz, USA), ERK (anti-mouse, 1:500, Cell Signaling Technology, American), p-ERK (anti-mouse, 1:500, Cell Signaling Technology, American), AKT (anti-mouse, 1:500, Cell Signaling Technology, American), p-AKT (anti-mouse, 1:500, Cell Signaling Technology, American) and glyceraldehyde-3-phosphate dehydrogenase (GAPDH) (anti-mouse, 1:1000, Santa Cruz, USA). After washed with PBST containing 20% Tween-20, the membranes were incubated with goat anti-rabbit IgG conjugated to horseradish peroxidase (1:5000, Southern Biotech, USA). Immunoblots were visualized and quantified using an enhanced chemiluminescence system. Relative protein levels were normalized to GAPDH, which was used as a loading control for total protein.

### Measurement of lactate and ATP

The Lumbar SC (L4–L5) were excised rapidly from deeply anesthetized rats, and then the tissues were homogenized into 500 μl lactate assay buffer (Lactate Colorimetric kit, Abcam) and centrifuged at 10,000×*g* for 4 min. Samples were tested according to the manufacturer’s protocol and lactate levels were normalized to control samples. ATP levels were measured using a ATP assay kit (Beyotime, China) according to the manufacturer’s instructions. All operations were performed on ice to precisely determine the ATP concentration.

### Enzyme linked immunosorbent assay (ELISA)

Protein samples were prepared in the same way as Western blot. Levels of TNF-α and IL-1β in each group were detected by ELISA kits (Jiancheng Biotech, Nanjing, Jiangsu, China) according to the manufacturer’s instructions.

### Immunohistochemistry

Under deep anesthesia with pentobarbital sodium, rats were transcardially perfused with 0.9% saline followed by 4% paraformaldehyde. The L4-L5 SC were dissected out and post-fixed in 4% paraformaldehyde overnight at 4 °C. After consecutively dehydrated in 20% and 30% sucrose, SC sections were crosscut into 8 um thick in a cryostat and blocked with 10% donkey serum, 3% bovine serum albumin and 0.3% Triton X-100 for 2 h at room temperature. Then, the sections were incubated with the following primary antibodies overnight at 4 °C: PKM-2(anti-mouse, 1:50, Santa Cruz, USA), neuronal nuclei (NeuN) (anti-rabbit, 1:300, Cell Signaling Technology, American), glial fibrillary acidic protein (GFAP) (anti-rabbit, 1:200; Sigma, USA) and ionized calcium-binding adapter molecule 1 (Iba1) (anti-rabbit, 1:500, Wako, Japan). The sections were incubated with FITC-conjugated Donkey or CY3-conjugated Donkey secondary antibodies or a mixture of both for double staining. After washed three times in PBS, the sections were examined with a Leica fluorescence microscope.

### Statistical analyses

Data were analyzed using SPSS 22.0 software and results were expressed as means ± SEM. Image J was used to process the density of specific bands and fluorescence intensity. Behavioral date was analyzed by a two-way repeated measures analysis of variance followed by Bonferroni test as the multiple comparison analysis. Differences between two groups were analyzed with Student t test. A value of *p* < 0.05 was considered statistically significant.

## Results

### CCI produced neuropathic pain accompanied by the upregulation of PKM2 in SC

**Fig. 1:**
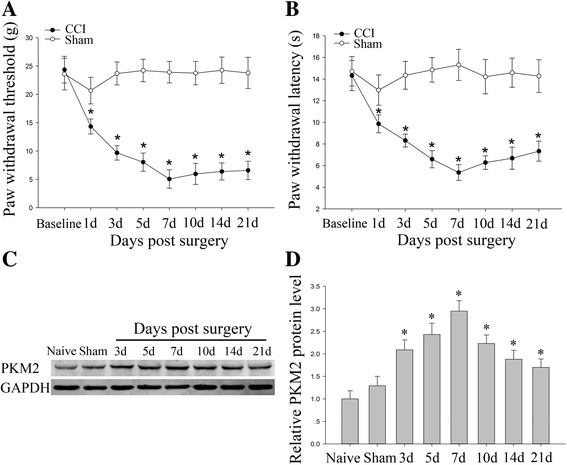
Changes of mechanical allodynia, heat hyperalgesia and PKM2 expression in rats after CCI. **a**, **b** CCI induced a significant decrease in PWT (**a**) and PWL (**b**). *P < 0.05 versus sham group. Five rats per group. **c**, **d** Western blot was performed to detect PKM2 expression in spinal cord. Each group comprised four corresponding spinal cord segments. Representative PKM2 protein bands were exhibited on the left (**c**), and statistical analysis histogram was shown on the right (**d**). *P < 0.05 versus sham group

As is shown in Fig. 1a, b, there were no statistical differences in PWT or PWL between groups 1 day before surgery (*p* > 0.05). In CCI group, PWT and PWL decreased at day 1 after CCI and then gradually reduced to the minimum at day 7 and maintained at a low level until day 21 compared with sham group (*p* < 0.05) (Fig. 1a, b). These behavioral changes suggested that CCI produced a progressive development of neuropathic pain. Western blot analysis showed that CCI rapidly and persistently increased PKM2 expression in SC compared with naïve rats (*P* < 0.05), starting at day 3, peaking at day 7 and maintaining until day 21 (Fig. 1c, d). However, sham surgery had no significant effect on PKM2 expression in SC at day 7 as compared to naïve rats (*p* > 0.05).

### CCI enhanced PKM2 expression in neurons and glia cells

**Fig. 2:**
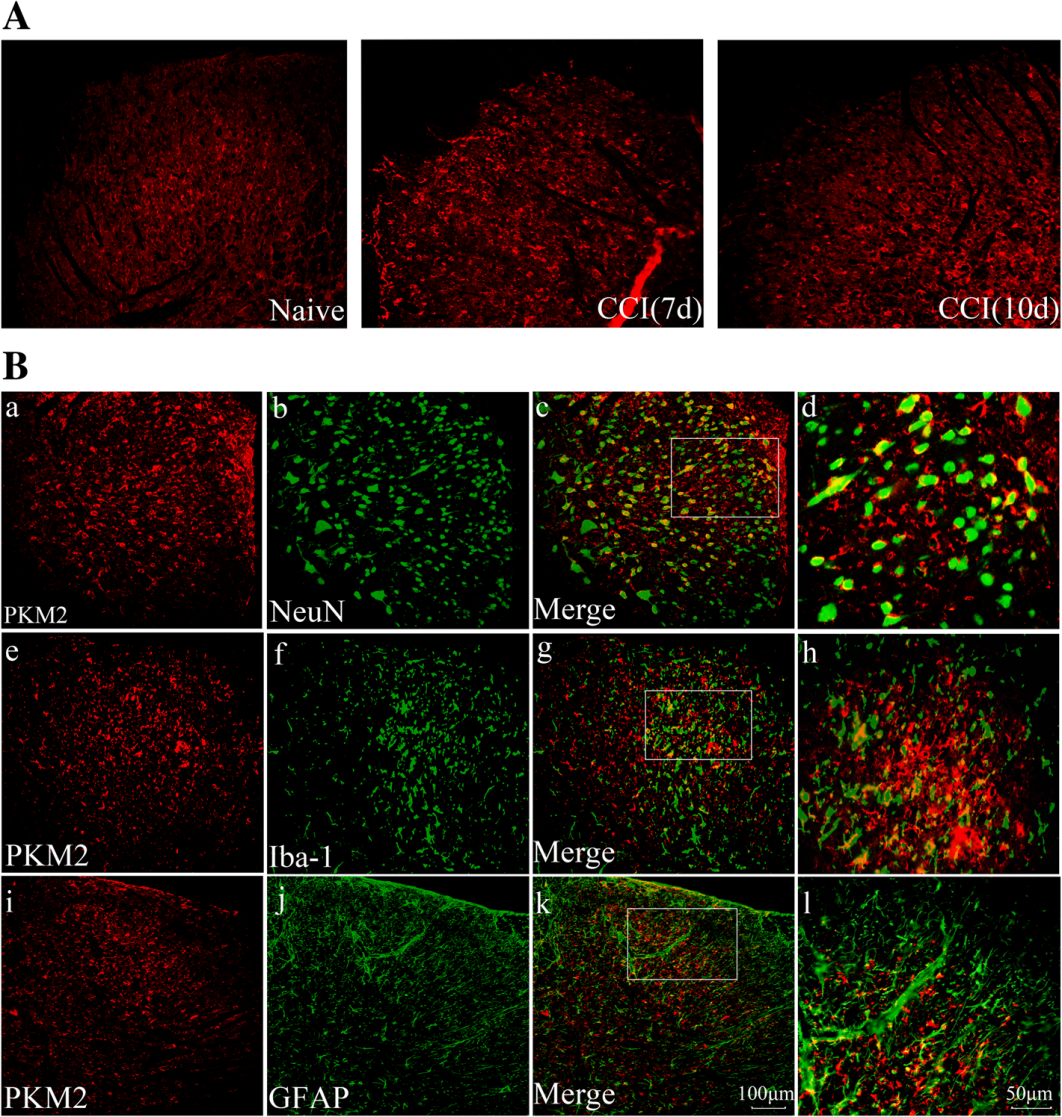
Expression and distribution of PKM2 in spinal cord dorsal horn after CCI. **A** Immunofluorescence showed that expression of PKM2 (red) was increased at day 7 and day 10. **B** Double staining showed that PKM2 co-localized with neuron marker NeuN (a-d), microglia marker Iba-1 (e-h), and astrocyte marker GFAP (i-l)

We then invested the expression and cellular distribution of PKM2 in SC dorsal horn after CCI by immunofluorescence staining. As is shown in Fig. 2a, PKM2 presented low basal expression level in ipsilateral SC dorsal horn in naïve animals. However, a marked increase of PKM2 immunoreactivity at day 7 and day 10 could be observed in CCI rats (Fig. 2a). We performed double staining of PKM2 with three major spinal nerve cell-specific markers at day 7: NeuN (for neurons), GFAP (for astrocytes) and Iba-1(for microglia). As is shown in Fig. 2b, PKM2 co-localized with NeuN, GFAP and Iba-1. These results suggested that PKM2 widely expressed in neurons, astrocytes and microglia cells after CCI.

### PKM2 siRNA attenuated not only pain hypersensitivity but also the production of lactate, TNF-α and IL-1β in SC after CCI

**Fig. 3:**
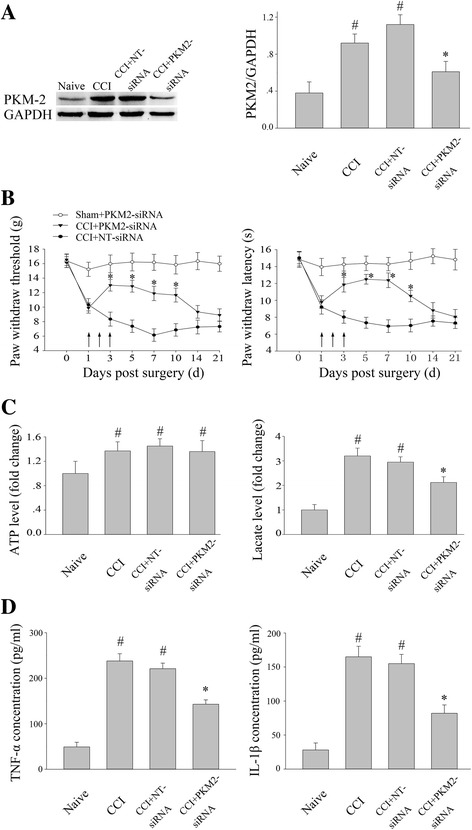
Intrathecal PKM2 siRNA treatment attenuated pain hypersensitivity and the production of lactate and pro-inflammatory transmitter induced by CCI. **a** The expression of PKM2 following intrathecal injection of siRNAs after CCI; PKM2 siRNA and NT siRNA (daily for 3 consecutive days, 15 μl each time, 0.75 μg/μl) were intrathecally injected. Tissues were harvested at day 3, 6 h after the last injection. *P < 0.05 versus NT siRNA group, ^#^*P* < 0.05 versus naïve group. Four rats per group. **b** Intrathecal injection of PKM2 siRNA for three consecutive days (day 1, 2, 3) increased paw withdraw threshold and latency for almost 7 days (day 3 to day 10). **c** PKM2 siRNA significantly attenuated lactate production, while ATP production were not influenced. **d** Meanwhile, both TNF-α and IL-1β production were also inhibited by PKM2 siRNA. *P < 0.05 versus NT siRNA group. ^#^*P* < 0.05 versus naïve group. Four rats per group

To investigate the function of PKM2 in neuropathic pain, we performed intrathecal injection of 15 μl PKM2 siRNA or NT siRNA (0.75 μg/μl) for three continuous days (day 1, 2, 3) after CCI. Western blot analysis was used to confirm the knock-down efficiency at day 3, 6 h after the last injection. As is shown in Fig. 3a, PKM2 siRNA (*P* < 0.05) significantly decreased PKM2 expression, while NT-SiRNA didn’t. PKM2 siRNA partially restrained the downtrend of PWT and PWL for approximately 7 days in CCI rats (*P* < 0.05) (Fig. 3b). However, PKM2 siRNA did not affect normal pain sensation in sham group (*P* > 0.05) (Fig. 3b). CCI induced significant increase in ATP and lactate level in L4-5 SC (*P* < 0.05) (Fig. 3c). The increased lactate level was inhibited by PKM2 siRNA (*P* < 0.05), while the ATP level was not influenced (*P* > 0.05) (Fig. 3c). Besides, in view of the important role of PKM-2 in the process of inflammation, we performed ELISA to exam the change of inflammatory factors in SC. CCI induced notable increase in TNF-α and IL-1β in SC (*P* < 0.05), and the increase was significantly inhibited by PKM-2 siRNA (*P* < 0.05) (Fig. 3d).

### Reducing PKM2 expression inhibited the activation of STAT3 and ERK signaling induced by CCI

**Fig. 4:**
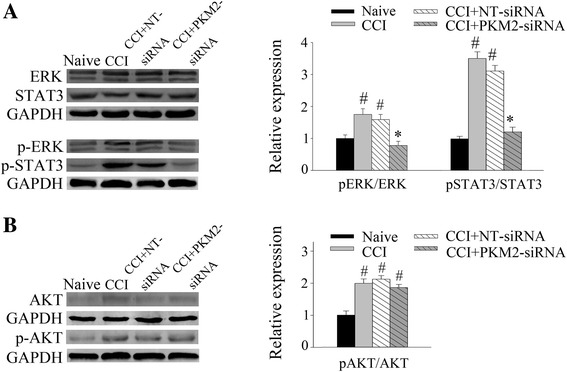
PKM2 siRNA reversed the phosphorylation of ERK and STAT3 in the spinal cord L4–5 segments induced by CCI. Western blot was performed to evaluate the levels of phosphorylated ERK, STAT3, and AKT in protein extracted from the L4–5 segment of spinal cord in each group. GAPDH was used as an internal control. **a** Treatment with PKM2 siRNA significantly reduced the pERK/ERK and pSTAT3/STAT3 ratios. *P < 0.05 versus NT siRNA group. ^#^*P* < 0.05 versus naïve group. Four rats per group. **b** PKM2 siRNA failed to inhibit the phosphorylation of AKT. ^#^*P* < 0.05 versus naïve group. Four rats per group

In an effort to clarify the potential mechanism of PKM2 in the process of neuropathic pain, we examined the phosphorylation level of several classic signaling pathways in neuropathic pain model after treatment with PKM2 siRNA. Increased  PKM2 in CCI group was coincided with elevated pSTAT3/STAT3 (*P* < 0.05), pERK/ERK (*P* < 0.05) and pAKT/AKT (*P* < 0.05) expression (Fig. 4a, b). PKM2 siRNA treatment significantly reduced the pSTAT3/STAT3 (*P* < 0.05) and pERK/ERK (*P* < 0.05) ratios (Fig. 4a). However, the pAKT/AKT ratio was not influenced by PKM2 siRNA (*p* > 0.05) (Fig. 4b).

## Discussion

In present study, we investigated the analgesic effect of intrathecal injection of PKM2 siRNA in a neuropathic pain animal model induced by sciatic nerve ligation. An appropriate animal model is important for the preclinical study of pain. We established CCI model by ligating sciatic nerve as described by Ding Y [32] for mirroring features of clinical neuropathic pain. The progressive mechanical allodynia and thermal hyperalgesia, which were characterized by the reduction of PWT and PWL, indicated that we successfully established the neuropathic pain model.

After peripheral nerve injury, glial cells were activated immediately and secreted massive inflammatory and neurotransmitter mediators, which sensitized neuron and furtherly aggravated central sensitization [35, 36]. Sensitized neuron and activated glial cells underwent metabolic changes to meet the enhanced energy demand and to respond to the disorders of nervous system better [13, 37]. The alterations of metabolism promoted pro-inflammatory phenotype conversion, transcription regulation, as well as posttranscriptional events in these immune cells [38]. In previous studies, CCI produced significant increases in glucose utilization and metabolic rate in spinal dorsal horns [39, 40]. Decades of researches have shown that PKM2 seems to be an important linker between metabolism and inflammation [38, 41]. In a proteomics study, Komori et al. found that PKM2 was significantly upregulated in dorsal root ganglions in a ligation spinal nerve induced-neuropathic pain model [42]. Hence, whether PKM2 participates in neuropathic pain has caught our most attention. In our study, we found that PKM2 quickly increased in SC after CCI, accompanied by behavioral changes. It indicates that PKM2 may participate in the process of neuropathic pain. To prove our speculation, we performed intrathecal injection of PKM2 siRNA in rats. Our results showed that siRNA not only suppressed the PKM2 protein expression, but also obviously alleviated CCI induced pain hypersensitivity. These data suggested that PKM2 plays a critical role in the development of neuropathic pain.

To explore the detailed mechanisms by which PKM2 contributed to neuropathic pain, we then investigated the downstream events of PKM2. PKM2 exists in two forms-- enzymatic active tetramer and protein kinase active dimeric. The tetramer form drives glucose towards oxidative metabolism via the tricarboxylic acid cycle, accompanied by the generation of large amounts of ATP [43]. Although the dimers form of PKM2 does not exert metabolic enzyme activity, it can regulate cell metabolism via its non-metabolic functions [44]. When epidermal growth factor receptor and platelet-derived growth factor receptor are activated, the dimeric PKM2 translocates into the nucleus and interacts with hypoxia inducible factor-1 and β-catenin to promote the expression of target genes, including GLUT1, SLC2A1, LDHA and PDK1. Upregulation of these glycolysis genes enhances glucose consumption and lactate production [44, 45]. ATP and lactate are important energy substance [19] involved in central sensitization and mediate allodynia in multiple pain models [20, 21]. In our study, we observed increases in ATP and lactate in SC. Interestingly, lactate significantly decreased after intrathecal injection of PKM2 siRNA, while the ATP concentration was not obviously affected.

In addition to the well-known role in glucose metabolism, PKM2 can also translocate into the nucleus in a dimeric form and function as protein kinase to regulate gene transcription under certain pathophysiological conditions. Nuclear PKM2 can phosphorylate the transcription factor STAT3, ERK1/2 and PI3K/ATK signaling pathway to promote cell proliferation and invasion [24, 26, 27]. Additionally, PKM2 increased strongly in LPS-activated macrophages, translocated into the nucleus and bounded with HIF-1α and STAT3 to promote the expression of inflammatory cytokines Il-1β and TNF-α [31, 46]. Inhibiting these signaling pathways can effectively suppress the generation of pro-inflammatory and neuropathic pain [28–30]. Accordingly, we examined the phosphorylation of these signaling pathways after intrathecal injection of PKM2 siRNA. PKM2 siRNA inhibited the phosphorylation of STAT3 and ERK signaling, but did not influence AKT signaling. Furthermore, IL-1β and TNF-α were down-regulated in SC. These results indicated that PKM2 contributes to neuropathic pain and inflammatory responses, possibly through regulating ERK and STAT3 signaling pathway as a protein kinase.

Furthermore, it is worth noting that oxidative stress plays an important role in central sensitization [47]. Reducing oxidative stress level by inhibiting nuclear factor E2-related factor 2, a critical endogenous protective factor in antioxidant defense, can effectively alleviate neuropathic pain [48]. Under stimuli of oxidative stress, PKM2 translocates into nucleus to function as a protein kinase [49]. So, weather PKM2 is involved in oxidative stress during the process of neuropathic pain needs further study in future.

## Conclusion

In conclusion, the present study demonstrated that CCI induced significant increase of PKM2 in SC. RNAi-mediated down-regulation of PKM2 effectively attenuated CCI-induced rat neuropathic pain and inflammatory responses, possibly through regulating ERK and STAT3 signaling pathway. Therefore, reducing PKM2 levels in SC using siRNA might be an effective therapeutic approach for relieving neuropathic pain.

## Abbreviations

ATP: Adenosine triphosphate
CCI: Chronic constriction injury
ELISA: Enzyme linked immunosorbent assay
ERK: Extracellular regulated protein kinases
GAPDH: Glyceraldehyde-3-phosphate dehydrogenase
GFAP: Glial fibrillary acidic protein
HIF-1α: Hypoxia-inducible factor 1-alpha
Iba-1: Ionized calcium-binding adapter molecule 1
IL-1β: Interleukin-1 beta
NeuN: Neuronal nuclei
NT-siRNA: Non-targeting siRNA
PKM2: Pyruvate kinase isozymes M2
PL3K/AKT: Phosphoinositide 3-kinase/protein kinase inhibitor
PWL: Paw-withdrawal latency
PWT: Paw withdrawal threshold
SC: Spinal cord
siRNA: Small interfering RNA
STAT3: Signal transducers and activators of transcription 3
TNF-α: Tumor necrosis factor alpha
